# Ascorbic acid 2-glucoside preconditioning enhances the ability of bone marrow mesenchymal stem cells in promoting wound healing

**DOI:** 10.1186/s13287-022-02797-0

**Published:** 2022-03-21

**Authors:** Yi Yi, Min Wu, Xiaomei Zhou, Mingchen Xiong, Yufang Tan, Honghao Yu, Zeming Liu, Yiping Wu, Qi Zhang

**Affiliations:** Department of Plastic Surgery, Tongji Hospital, Tongji Medical College, Huazhong University of Science and Technology, 1095 Jiefang Avenue, Wuhan, 430030 Hubei China

**Keywords:** Wound healing, BMSCs, AA2G, Angiogenesis, Collagen formation

## Abstract

**Background:**

Nowadays, wound is associated with a complicated repairing process and still represents a significant biomedical burden worldwide. Bone marrow mesenchymal stem cells (BMSCs) possess multidirectional differentiation potential and secretory function, emerging as potential cellular candidates in treating wounds. Ascorbic acid 2-glucoside (AA2G) is a well-known antioxidant and its function in BMSC-promoting wound healing is worth exploring.

**Methods:**

The in vitro cell proliferation, migration, and angiogenesis of BMSCs and AA2G-treated BMSCs were detected by flow cytometry, EDU staining, scratch assay, transwell assay, and immunofluorescence (IF). Besides, the collagen formation effect of AA2G-treated BMSCs conditioned medium (CM) on NIH-3T3 cells was evaluated by hydroxyproline, qRT-PCR and IF staining detection. Next, in the wound healing mouse model, the histological evaluation of wound tissue in PBS, BMSCs, and AA2G-treated BMSCs group were further investigated. Lastly, western blot and ELISA were used to detect the expression levels of 5-hmc, TET2 and VEGF protein, and PI3K/AKT pathway activation in BMSCs treated with or without AA2G.

**Results:**

The in vitro results indicated that AA2G-treated BMSCs exhibited stronger proliferation and improved the angiogenesis ability of vascular endothelial cells. In addition, the AA2G-treated BMSCs CM enhanced migration and collagen formation of NIH-3T3 cells. In vivo, the AA2G-treated BMSCs group had a faster wound healing rate and a higher degree of vascularization in the new wound, compared with the PBS and BMSCs group. Moreover, AA2G preconditioning might enhance the demethylation process of BMSCs by regulating TET2 and up-regulating VEGF expression by activating the PI3K/AKT pathway.

**Conclusions:**

AA2G-treated BMSCs promoted wound healing by promoting angiogenesis and collagen deposition, thereby providing a feasible strategy to reinforce the biofunctionability of BMSCs in treating wounds.

**Supplementary Information:**

The online version contains supplementary material available at 10.1186/s13287-022-02797-0.

## Introduction

Wound healing is a complex repair process involving multiple cell types, extracellular matrix, and soluble factors that contribute to skin regeneration after injury [1]. Generally speaking, the healing process consists of four temporally ordered and overlapping phases, including hemostasis, inflammation, proliferation, and tissue remodeling [2]. Disorders at any stage of the wound healing process could lead to the formation of various refractory ulcers or excessive scarring. Therefore, wound healing is a key issue of clinical concern, but it is still confronted with difficult problems, such as skin irritants, poor repair, and scar formation.

Bone marrow mesenchymal stem cells (BMSCs) are multilineage cells with the capability to self-renew, multipotential differentiation, and secretion of various biological active factors, thus possessing great potential in regenerative medicine [3]. BMSCs transplantation has been acknowledged as a promising therapeutic strategy for wound healing, probably due to the BMSC ability of multidirectional differentiation, low immunogenic property, and multiple growth-promoting factor release [4]. Existing studies have shown that BMSCs have been endowed with great application prospects in various animal wound healing models, but not in clinical practice [5]. The limited migration ability, survival rate, and secretion function of BMSCs are the limiting factors for their potential application. Therefore, many studies have investigated that specific gene modification and intervention preconditioning of BMSCs could enhance their effectiveness in accelerating wound healing. For example, Xia et al. reported that the hVEGF(165)/hBD3-modified BMSCs markedly promoted wound healing by enhancing the expression of hVEGF(165) and hBD3 [6]. Zhang et al. also verified that activin B prominently promoted the BMSC-mediated cutaneous wound healing by controlling cell migration through the JNK-ERK signaling pathway [7].

Ascorbic acid 2-glucoside (AA2G), a glycosylated ascorbic acid (AA), is a stable derivative of Vitamin C (VitC) [8]. AA2G is a well-known antioxidant with excellent antioxidant properties and bioavailability. AA2G can act as a radical scavenger and decrease initial DNA damage, thereby protecting cells from radiation [9]. The chrysanthemum morifolium flower extract (Chrys) and AA2G, could synergistically offer prolonged protection against ROS and melanin formation, and UVA-mediated DNA damages [10]. AA2G has a protective effect against helicobacter pylori infection in gastric epithelial cells [11]. Of particular, Lee et al. proved that AA2G conferred to the stability of naive pluripotency in mouse embryonic stem cells (mESCs) and primordiousness of human mesenchymal stem cells (hMSCs) without cellular toxicity [12]. Furthermore, the supplementation of hMSCs with AA2G significantly improved the treatment outcome of asthmatic models in vivo, by promoting self-renewal, engraftment, and anti-inflammatory properties of hMSCs [12].

Considering the positive protective effect of AA2G, it will be a valuable proposition to explore the AA2G potential application in sustaining the BMSC stemness, developmental potency, and therapeutic efficacy in wound healing. It will be of particular intriguing to explore the role and mechanism of AA2G-treated BMSCs in treating wound healing. To address this issue, we initially investigated the impact of AA2G-preconditioned BMSCs on the proliferation, migration, and angiogenesis, and then evaluated the effect of AA2G-preconditioned BMSCs on migration and collagen capacity of NIH-3T3 cells. Lastly, we also investigated the ability of AA2G-preconditioned BMSCs in full-thickness wound healing in mice. Our results collectively confirmed that AA2G-preconditioned BMSCs accelerated the process of wound healing via promoting angiogenesis and collagen deposition.

## Materials and methods

### Mouse BMSCs isolation and identification

Primary BMSCs were extracted from BALB/C mice (3 weeks, male) as previously described [13]. After killing by neck dislocation under anesthesia, these mice were immersed in 75% alcohol for 15 min. Under sterile conditions, both femurs were quickly removed and the bone marrow cavity was rinsed with culture solution containing Dulbecco’s Modified Eagle Medium/Nutrient Mixture F-12 (DMEM/F12) (Gibco, USA) to collect bone marrow cell suspension. After centrifugation, the cell suspension was were filtered through a 200-mesh filter, inoculated and cultured in a culture flask (37 °C and 5% CO2). The cell culture system has consisted of DMEM/F12, 20% fetal bovine serum (FBS) (Gibco, USA), 100 U/mL penicillin and 100 μg/mL streptomycin. For biomarker identification, the single-cell suspension of BMSCs was prepared and was stained with CD29, CD44, CD90, CD31, and CD34 antibodies (Becton Dickinson, USA) for 30 min, respectively. The stained samples were washed with PBS and then suspended for analysis of flow cytometry (FCM) (Becton Dickinson, USA). BMSCs from passage 3 to 7 were collected for subsequent experiments. To identify the adipogenesis function, the BMSCs were cultured with an inducible mixture of DMEM/F12 with 10% FBS, 0.5 μM dexamethasone, 50 μM isobutylmethylxanthine (IBMX), 10 μg/mL insulin, and 50 μM indomethacin for 28 d, as previously reported [14]. Lastly, to identify the osteogenesis and chondrogenesis ability, BMSCs were respectively cultured in BMSC osteogenic differentiation medium and BMSC chondrogenic differentiation medium (Cyagen Biosciences, China), according to the manufacturer’s instructions.

### Mouse wound healing model establishment

A total of 45 BALB/C mice (8 weeks, male) were anesthetized by intraperitoneal injection of sodium pentobarbital (60 mg/kg). Full-thickness skin wound models were created in the middle of the back of mice using an 8 mm biopsy needle (Kai Medical, Solingen, Germany). Subsequently, the mice were randomly divided into three groups: control group (100 μL PBS, *n* = 15), BMSCs group (2 × 10^6^ BMSCs resuspended in 100 μl PBS, *n* = 15), AA2G-treated BMSCs group (2 × 10^6^ AA2G-treated BMSCs were resuspended in 100 μL PBS, *n* = 15). The 100 μL PBS suspensions were injected into 4 sites around the wound and 1 site in the center of the wound. The wound closed areas were photographed and wound tissues were obtained at day 0, 3, 7, and 14 post-injection. The skin wound images were imported into Image J software (National Institutes of Health, USA) for wound healing assay. The wound closure rate was calculated using (W_0_—W_t_)/W0 × 100%, where W_0_ represented the initial wound area and W_t_ represented the wound area at the corresponding measurement point [15]. This animal experiment was approved by the ethical committee of Tongji Hospital.

### Cell culture

The mouse embryonic fibroblast NIH-3T3 and mouse vascular endothelial cells C166 were cultured by 90% DMEM (Gibco, USA) and 10% FBS (Gibco, USA). As previously reported, BMSCs were cultured in the medium containing 0.74 mM AA2G (Sigma-Aldrich, USA) for 3 days and named AA2G-treated BMSCs [16]. After 3 days of AA2G culture, the serum-free medium was used to continue culture these cells, and the according obtained conditioned medium (CM) was named AA2G-treated BMSC CM. At the same time, BMSCs were cultured in a serum-free medium, and the according obtained CM was named BMSC CM. The NIH-3T3 cells were treated with AA2G-treated BMSC CM and BMSC CM for 24 h respectively for detecting the migration ability and collagen production. For tube formation detection, matrigel (BD Biosciences, USA) was thawed and then transferred to a 24-well culture plate for solidification. C166 cells were inoculated onto matrigel and were treated with BMSC CM or AA2G-treated BMSC CM for 24 h at 37 °C. The images of tube formation were obtained by SOPTOP CX40 microscope (Shanghai, China).

### Cell proliferation

After 72 h of AA2G treatment, BMSCs were rinsed, fixed with 70% ethanol at 4 °C, and resuspended with PBS. In addition, the propidium iodide (Sigma-Aldrich, USA) was added into cell suspension. Cell cycles in G1, S, and G2 were analyzed by FCM analysis. Besides, the EDU staining was performed according to the instructions of the EDU staining kit (Beyotime, China).

### Cell migration

BMSCs were inoculated into 6-well plates and divided into the control group and AA2G-treated BMSCs group. Meanwhile, NIH-3T3 cells were inoculated into six-well plates and divided into control group, BMSCs CM, and AA2G-treated CM groups. When the cells grew to 90% confluence, 200 μL microtubule tips were used to create scratches, while the medium was replaced with a serum-free medium. The wounds were photographed at 0, 12, and 24 h after injury. Then, the wound widths and wound closure rates were measured by Image J software.

Transwell migration assay was performed with an 8-μm pore size Boyden chamber (Corning Costar, USA). BMSCs were placed in the top Boyden chamber and AA2G (final concentration 0.74 mM)was added in the lower chamber. In another transwell migration assay, NIH-3T3 cells were placed on the top Boyden chamber and AA2G-treated BMSC CM and BMSC CM were added to the lower compartment, respectively. After 48 h culture, the submembrane migration cells were fixed with 4% paraformaldehyde for 15 min and stained with 0.5% crystal violet. Migrating cells were observed under a SOPTOP CX40 microscope (Shanghai, China), and the number of migrating cells was determined by calculating the OD value of crystal violet staining.

### Quantitative real-time polymerase chain reaction (qRT-PCR)

RNAiso Plus (Takara Biomedical Technology, China) was used to extract total RNA from cell samples. Then, according to the manufacturer’s instructions, Hieff first-strand cDNA Synthesis Super Mix (Yeasen, China) was utilized to obtain the stable cDNA. SYBR Green real-time PCR Master Mix (Yeasen, China) was used to accurately evaluate mRNA expression. By using β-actin as an internal reference, the expression of related genes was calculated according to formula 2^−(△△CT)^. The forward (5’ to 3’) and reversed (5’ to 3’) primer sequences were shown as below:

β-actin: GGCTGTATTCCCCTCCATCG, CCAGTTGGTAACAATGCCATGT; MMP3: ACATGGAGACTTTGTCCCTTTTG, TTGGCTGAGTGGTAGAGTCCC; MMP9: GCAGAGGCATACTTGTACCG, GCAGAGGCATACTTGTACCG; VEGF: CTGCCGTCCGATTGAGACC, CCCCTCCTTGTACCACTGTC; HIF-1: ACCTTCATCGGAAACTCCAAAG, CTGTTAGGCTGGGAAAAGTTAGG;

### Western blotting

The protein expressions were detected by western blotting. Cell samples were homogenized with cell lysis buffer (Cell Signaling Technology, USA) and the supernatant was collected centrifugally. The samples were separated on 10% or 8% polyacrylamide-SDS gel and electrically imprinted on polyvinylidene fluoride (PVDF) membranes (Bio-Rad, USA). Then, the PVDF membranes were blocked in 5% non-fat milk in tris-buffered saline with Tween 20 (TBST) for 1 h and incubated with primary antibodies at 4 °C overnight. After washing, the PVDF membranes were incubated with the secondary HRP conjugated secondary antibodies (Proteintech, USA) at 1:5000 for 2 h at room temperature. Protein blots were detected by an ECL western blotting kit (Proteintech, USA) and quantitated by ImageLab software. The details of the primary antibodies in this experiment were presented as follows: anti-β-actin antibody (Proteintech, USA), anti-p-AKT antibody (Proteintech, USA), anti-AKT antibody (Proteintech, USA), anti-PIK3CA antibody (Abclonal, China), anti-p-PI3K antibody (Abclonal, China), anti-VEGF antibody (Proteintech, USA), and anti-TET2 antibody (Proteintech, USA).

### Histological examination

Mouse skin wound samples were fixed with 4% formalin solution and paraffin-embedded. The tissue sections were stained with hematoxylin and eosin (HE) and Masson trichrome respectively to detect the wound re-epithelization and collagen deposition. CD31 immunohistochemistry (IHC) staining was used to evaluate angiogenesis. Sections were incubated with anti-CD31 antibody (Proteintech, USA) and then incubated with peroxidase-conjugated secondary antibodies (Cell Signaling Technology, USA). The staining color was visualized using a DAB Peroxidase Substrate Kit (Maxin, China). The digital images were observed under the SOPTOP CX40 microscope (Shanghai, China).

### Immunofluorescence (IF) staining

Tissue sections and cells samples were fixed with 4% paraformaldehyde. After being sealed with 2% (W/V) bovine serum albumin (BSA) solution, the wound samples were incubated with primary antibody at 4 °C overnight, followed by incubation with according the second antibody at room temperature. The samples were restained with DAPI nuclear dye. The details of primary antibodies were as follows: anti-5-hmc antibody (Abcam, UK), anti-VEGF antibody (Proteintech, USA), anti-TGF-β antibody (Proteintech, USA).

### DNA demethylation analysis

The gene expression levels of 6 chip data were downloaded from the GEO database (GSE106184). The R packages “limma” and “clusterProfiler” were used to perform differentially expressed genes analysis and the Gene Ontology (GO) analysis, respectively. For ELISA analysis, the 5-hmc expression level of the BMSCs group and the AA2G-treated BMSCs group was quantified by a 5-hmc ELISA kit (Jiangsu Meibiao Biotechnology, China). All samples were tested following the instructions of the kit manufacturer.

### Statistical analysis

Two groups analysis was performed one-way analysis of variance with t-test by using Graphpad Prism 8.0 (GraphPad, USA). And three groups analysis was performed ANOVA with posthoc analysis by Bonferroni/Dunn’s test by using Graphpad Prism 8.0. Statistically significant was identified as *P* < 0.05.

## Results

### Identification of BMSCs

**Fig. 1:**
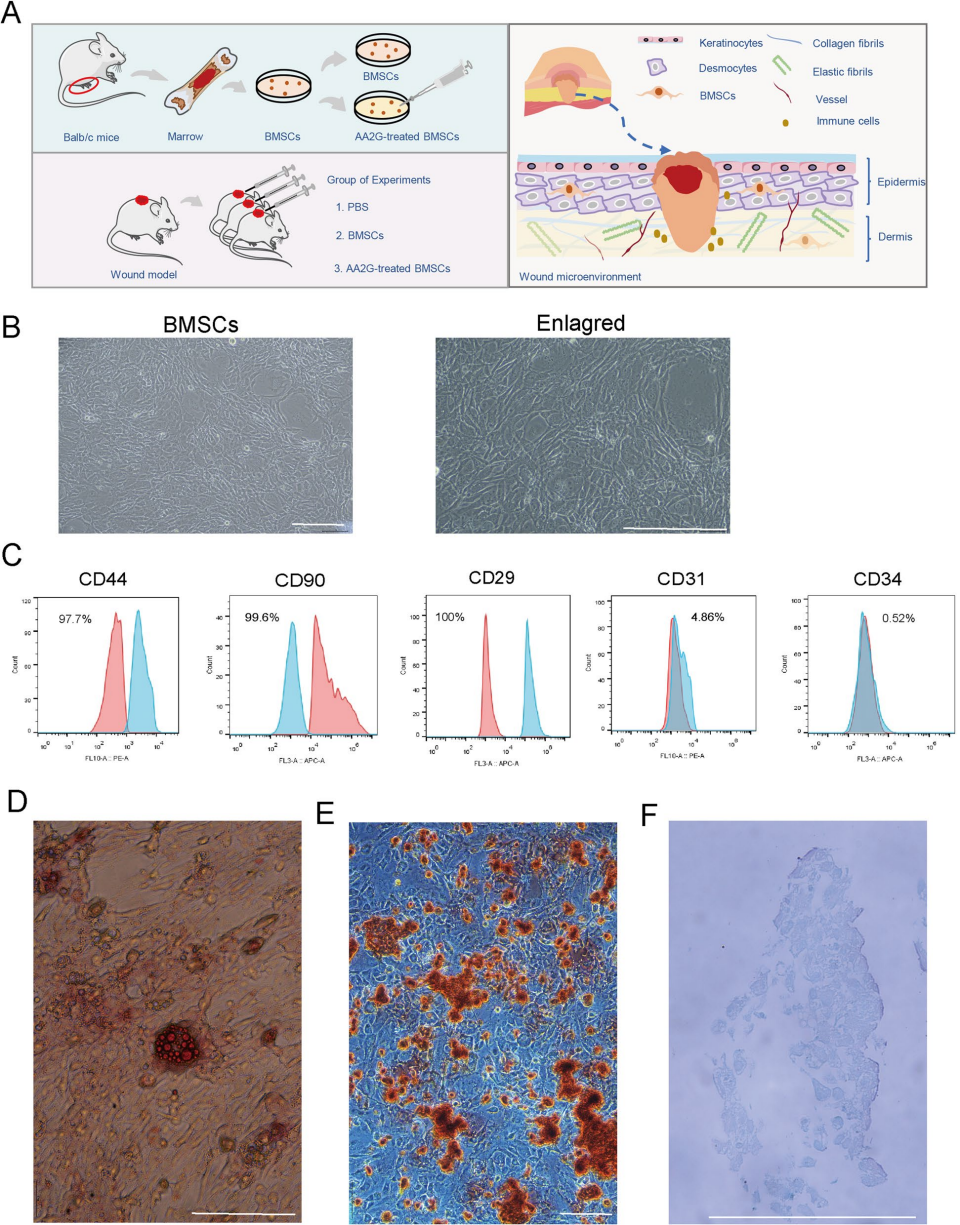
Identification of BMSCs. **a** Left part, the flow diagram of experimental. Wound mice were randomly divided into 3 groups that were treated with PBS, BMSCs, and AA2G-treated BMSCs, respectively. Right part, schematic diagram of tissue repair. Wound healing involves a variety of cellular interactions. **b** Morphology of BMSCs at passage 3. Bar = 120 µm. **c** FCM analysis of the expression of BMSCs surface markers, which were positive for CD44, CD90, and CD29 and negative for CD31 and CD34. **d** Oil-red O staining of adipogenesis differentiation of BMSCs. Bar = 120 µm. **e** Alizarin red staining of osteogenic differentiation of BMSCs. Bar = 120 µm. **f** Alcian blue staining of chondrogenic differentiation of BMSCs. Bar = 120 µm

The detailed study process was presented in Fig. 1a. The wound regeneration microenvironment is composed of multiple cell types (immune cells, stem cells, keratinocytes, desmocytes) and soluble factors, thus establishing a complex interaction network (right part, Fig. 1a). The bright-field images of the microscope showed that these BMSCs presented a spindle-shaped and fibroblast-like morphology (Fig. 1b). The FCM results emphasized that the surface biomarkers of obtained BMSCs were positive for MSC markers CD44, CD29, and CD90, but were negative for hematopoietic lineage marker CD34 and the endothelial marker CD31 (Fig. 1c, Additional file 1: Figure S1A). The Oil-red O staining results showed that the differentiated BMSCs possessed an expanded morphology, and contained large and lipid droplets within the cytoplasm, which was consistent with adipocyte features (Fig. 1d). Alizarin red staining was used to detect osteogenic differentiation of BMSCs. The results showed that the induced BMSCs were clustered and grew like colonies with red layered nodules of mineralization (Fig. 1e). Alcian blue staining was used to detect the accumulation of proteoglycan in cartilage, which showed that the induced BMSCs tend towards chondrogenic differentiation (Fig. 1f). Therefore, combined with morphology, biomarker evaluation, and multidirectional differentiation results, these separated cells could be identified as BMSCs.

### *AA2G treatment enhanced BMSC proliferation and migration *in vitro

**Fig. 2:**
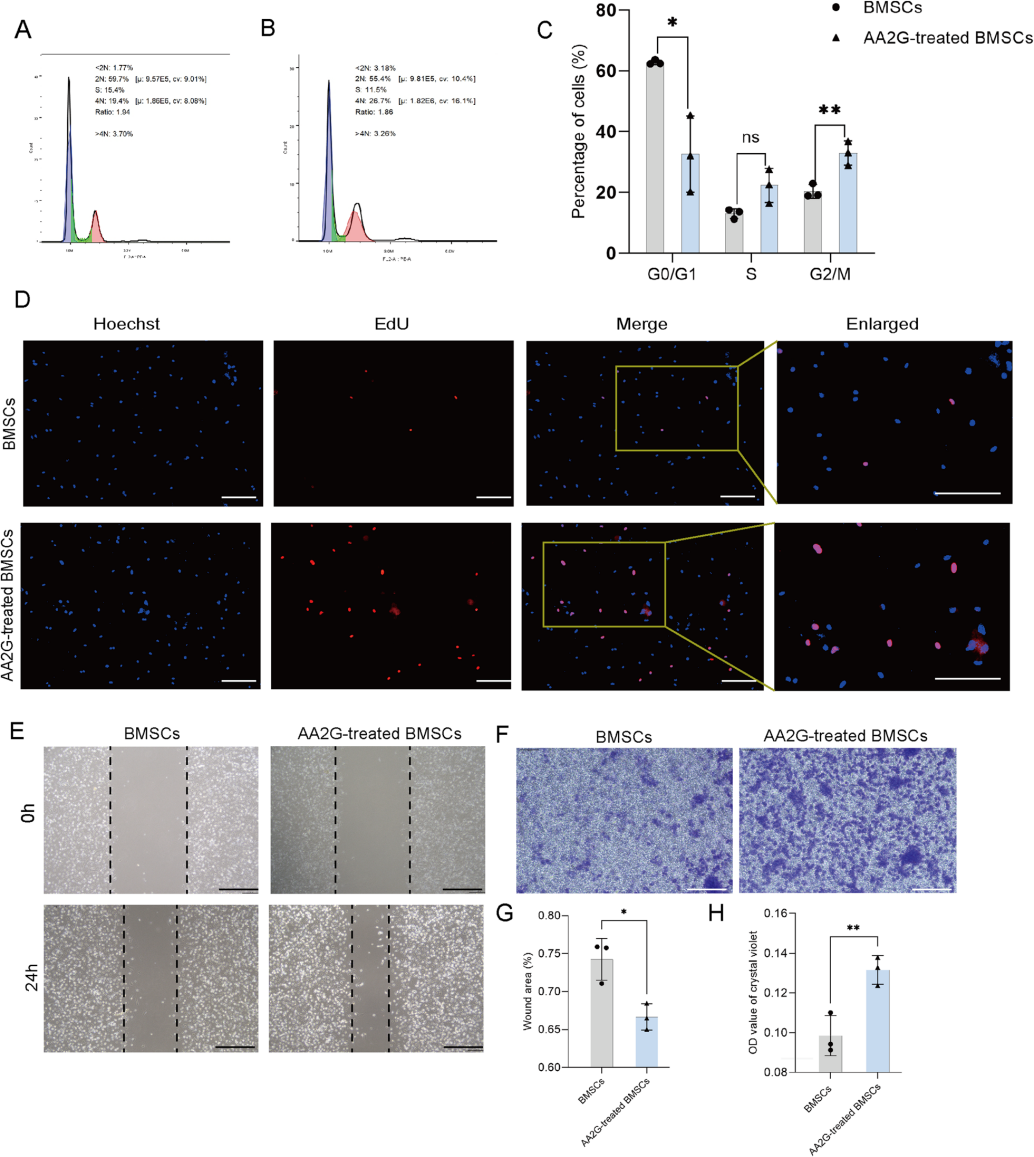
AA2G enhanced the proliferation and migration of BMSCs. **a**, **b** Representative images of the cell cycle by flow cytometry analysis. **c** Percentage of cell cycle distribution in BMSCs and AA2G-treated BMSCs group (*n* = 3). *indicated *P* < 0.05, **indicated *P* < 0.01, p-value based on t-test. **d** The cell proliferation in BMSCs and AA2G-treated BMSCs was detected by EDU staining. **e** Representative images of the scratch assay showed the migration of BMSCs and AA2G-treated BMSCs for 24 h. Bar = 120 µm. **f** Representative images of BMSCs metastasis stained with crystal violet. Bar = 120 µm. **g** Quantitative analysis of the percentage of scratch area in scratch wound healing assay (*n* = 3). *P* < 0.05 based on t-test. **h** Quantitative analysis of migrated cells in a transwell assay (*n* = 3). *P* < 0.05 based on t-test

The cell cycle assay and EDU assay were used to detect the effect of AA2G on the proliferation of BMSCs. FCM analysis showed that after treatment with AA2G, the distribution of BMSCs in the G1 phase was significantly lower than that in the control group, while the distribution of BMSCs in the G2 phase was significantly increased (Fig. 2a–c, Additional file 1: Figure S1B). Besides, the increase of positive EDU staining number in the AA2G-treated BMSCs group also suggested that AA2G treatment could promote BMSCs proliferation (Fig. 2d). These two assays together confirmed that AA2G might play a positive role in BMSC proliferation.

Moreover, scratch wound healing and transwell migration were performed to determine the effect of AA2G treatment in BMSC migration. At 24 h after scratch, AA2G-treated BMSCs migrated more to the scratch area compared with the BMSCs group (Fig. 2e, g). The transwell migration assay further indicated that the number of migrated cells in the AA2G-treated BMSCs group was significantly higher than that of the BMSCs group (Fig. 2f, h). Hence, these results suggested that AA2G possessed the ability to induce BMSC migration in vitro.

### AA2G-treated BMSCs promoted angiogenesis in vitro

**Fig. 3:**
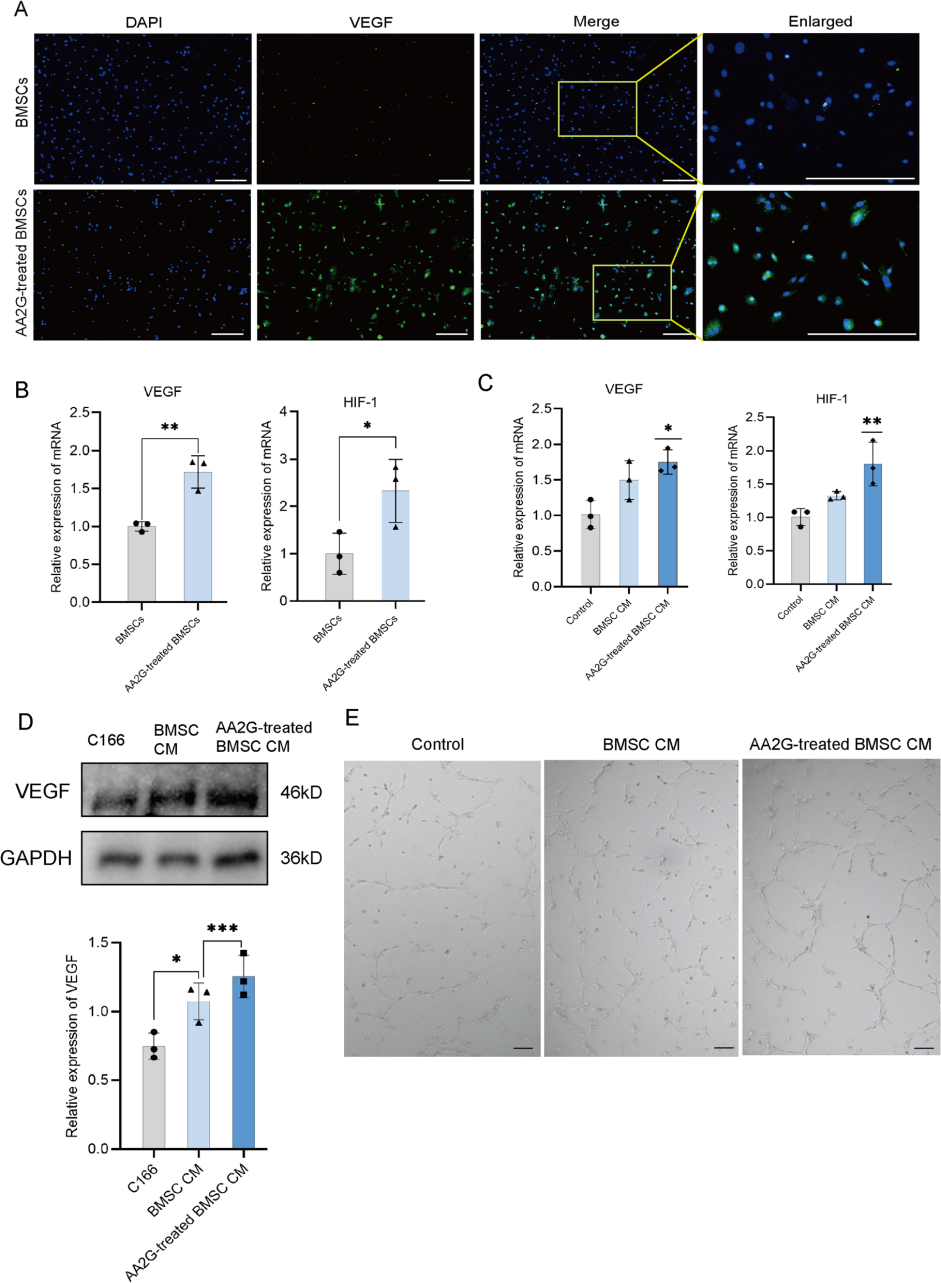
AA2G preconditioning in angiogenesis was associated with the AKT signaling pathway. **a** IF results showed the VEGF expression in BMSCs and AA2G-treated BMSCs. Bar = 120 µm. **b** qRT-PCR was used to detect the expression of VEGF and HIF-1 in BMSCs and AA2G-treated BMSCs (*n* = 3). * indicated *P* < 0.05, ** indicated *P* < 0.01, p-value based on t-test. **c** qRT-PCR was used to detect the expression of VEGF and HIF-1 in C166 cells (*n* = 3). * indicated *P* < 0.05, ** indicated *P* < 0.01, p-value based on t-test. **d** Western blot analysis and quantitative analysis of VEGF protein in C166 cells (*n* = 3). * indicated *P* < 0.05, *** indicated *P* < 0.001, p-value based on t-test. **e** Tube formation of C166 cells in the absence (control) or presence of BMSC or AA2G-treated BMSC CM. Bar = 200 µm

The effect of AA2G treatment on the vascularization ability of BMSCs is also the key event to wound healing. Firstly, the IF results showed that the positive expression of VEGF in the AA2G-treated BMSCs group was significantly higher than that in the BMSCs group (Fig. 3a). Then qRT-PCR results showed that AA2G significantly increased the mRNA expression levels of HIF-1 and VEGF in BMSCs (Fig. 3b). Meanwhile, in C166 cells, the qRT-PCR results also showed that the mRNA expression levels of HIF-1 and VEGF in the BMSC CM group were significantly higher than that in the control group, but their expressions in the AA2G-treated BMSC CM group were significantly higher than those in the other two groups (Fig. 3c). At the protein level, the expression of VEGF in the BMSC CM group was increased compared with the control group, and the expression of VEGF in the AA2G-treated BMSC CM group was higher than that of the BMSC CM group (Fig. 3d). In the tube forming experiment with C166 cells, compared with the other two groups, the number of tubules formed by endothelial cells was significantly increased in the AA2G-treated BMSC CM group (Fig. 3e). These results indicated that AA2G-treated BMSCs promoted angiogenesis in vitro.

### AA2G-treated BMSCs promoted NIH-3T3 cells migration and collagen formation in vitro

**Fig. 4:**
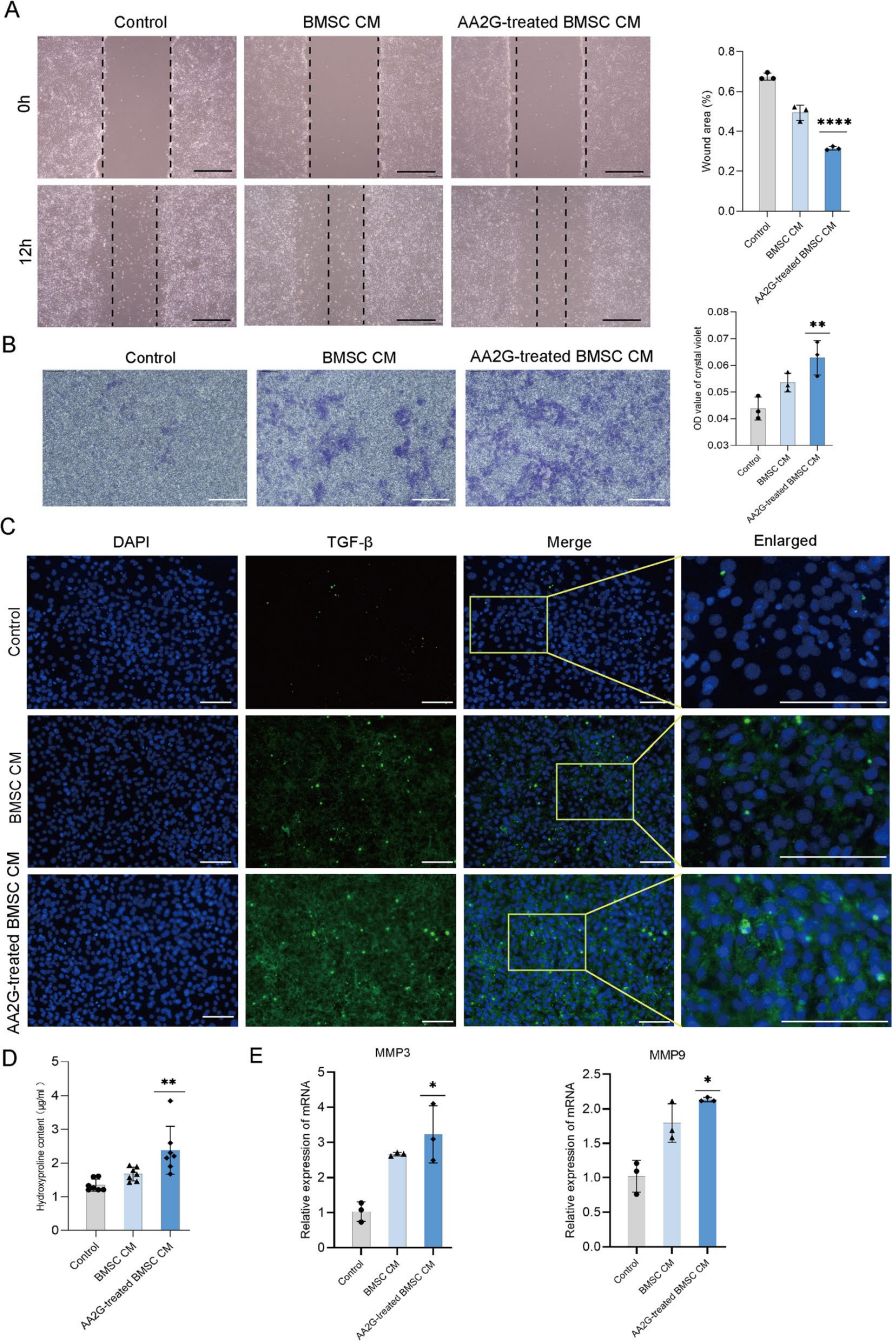
AA2G-treated BMSC CM promoted wound healing in vitro. **a** Scratch assay showed the migration capacities of NIH-3T3 cells treated with PBS, BMSC CM, and AA2G-treated BMSC CM for 12 h, respectively. Bar = 120 µm (*n* = 3). *P* < 0.0001 based on one-way ANOVA. **b** Images and quantitative analysis of transmigrated NIH-3T3 cells (stained with crystal violet) are shown. Bar = 120 µm (*n* = 3). *P* < 0.01 based on one-way ANOVA. **c** IF staining of TGF-β for NIH-3T3 treated with the above different CM respectively. Bar = 120 µm. **d** Hydroxyproline content in NIH-3T3 cells after different CM treatment for 24 h (*n* = 7). *P* < 0.01 based on one-way ANOVA. **e** qRT-PCR was used to detect the expression of collagen-related genes (MMP-3, MMP-9) in NIH-3T3 treated with different CM (*n* = 3). *P* < 0.05 based on one-way ANOVA

In the scratch assay, the migration cells in the inward scratch area of the AA2G-treated BMSC CM group were significantly more than those in the other two groups, indicating a higher wound healing rate (Fig. 4a). In the transwell migration assay, the number of downward ventricular migration cells in the AA2G-treated BMSC CM group was the largest among the three groups (Fig. 4b). As shown in Fig. 4c, the TGF-β positive cells in the AA2G-treated BMSC CM group were the most among the three groups. Hydroxyproline is an important component of collagen, and its content can reflect the synthesis of collagen. The hydroxyproline content detection showed that the hydroxyproline content in the AA2G-treated BMSC CM group was significantly increased compared with the control group and BMSC CM group (Fig. 4d). More intuitively, the expressions of MMP-3 and MMP-9 were also significantly increased in the AA2G-treated BMSC CM group, compared with the other two groups (Fig. 4e). These results suggested that the protective effect of AA2G-treated BMSCs on wound healing might be related to the positive regulation of fibroblasts, including promoting cell migration and collagen production.

### AA2G-treated BMSCs enhanced wound healing in mice

**Fig. 5:**
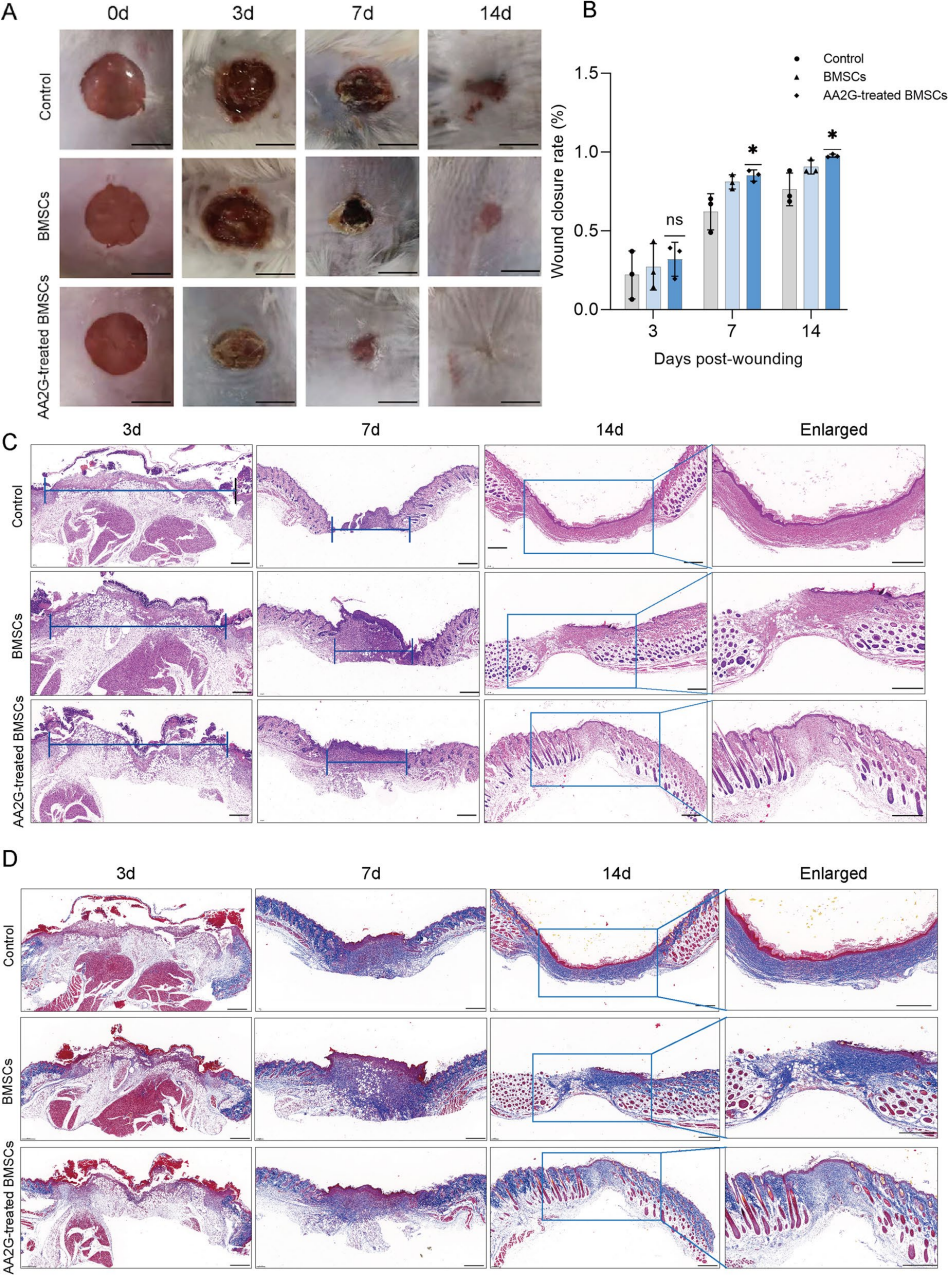
AA2G-treated BMSCs promoted wound healing in mice. **a** Overview of size changes of the wound made in the back skin of mice among the three groups at day 0, 3, 7, and 14 post-treatment. Bar = 5 mm. **b** The rate of wound closure after BMSCs or AA2G-treated BMSCs engrafting into the wounds (*n* = 3). *P* < 0.05 based on one-way ANOVA. **c** HE staining of wound granulation tissue in control, BMSCs, and AA2G-treated BMSCs groups at day 3, 7, and 14 post-wounding. The double-headed black arrows indicated the edges of the scars. Bar = 500 µm. **d** Masson staining of repaired tissue at day 3, 7, and 14 in control, BMSCs, and AA2G-treated BMSCs groups. Bar = 500 µm

In the wound healing mouse model, the wound healing rate of the BMSCs group was increased in mice at day 3, 7, and 14 compared with the control group, but the wound healing rate of the AA2G-treated BMSCs group was significantly better than that of the BMSCs group (Fig. 5a, b). Similarly, HE staining also showed that the cell density and epithelialization degree of granulation tissue in the AA2G-treated BMSCs group was increased significantly compared with the other two groups (Fig. 5c). In addition, collagen deposition was more orderly in the wound tissue of the BMSCs group and AA2G-treated BMSCs groups, but the collagen density was higher in the AA2G-treated BMSCs group (Fig. 5d). These results suggested that AA2G-treated BMSCs could accelerate wound healing by promoting collagen formation and epithelialization levels.

### AA2G-treated BMSCs enhanced angiogenesis in wound tissues

**Fig. 6:**
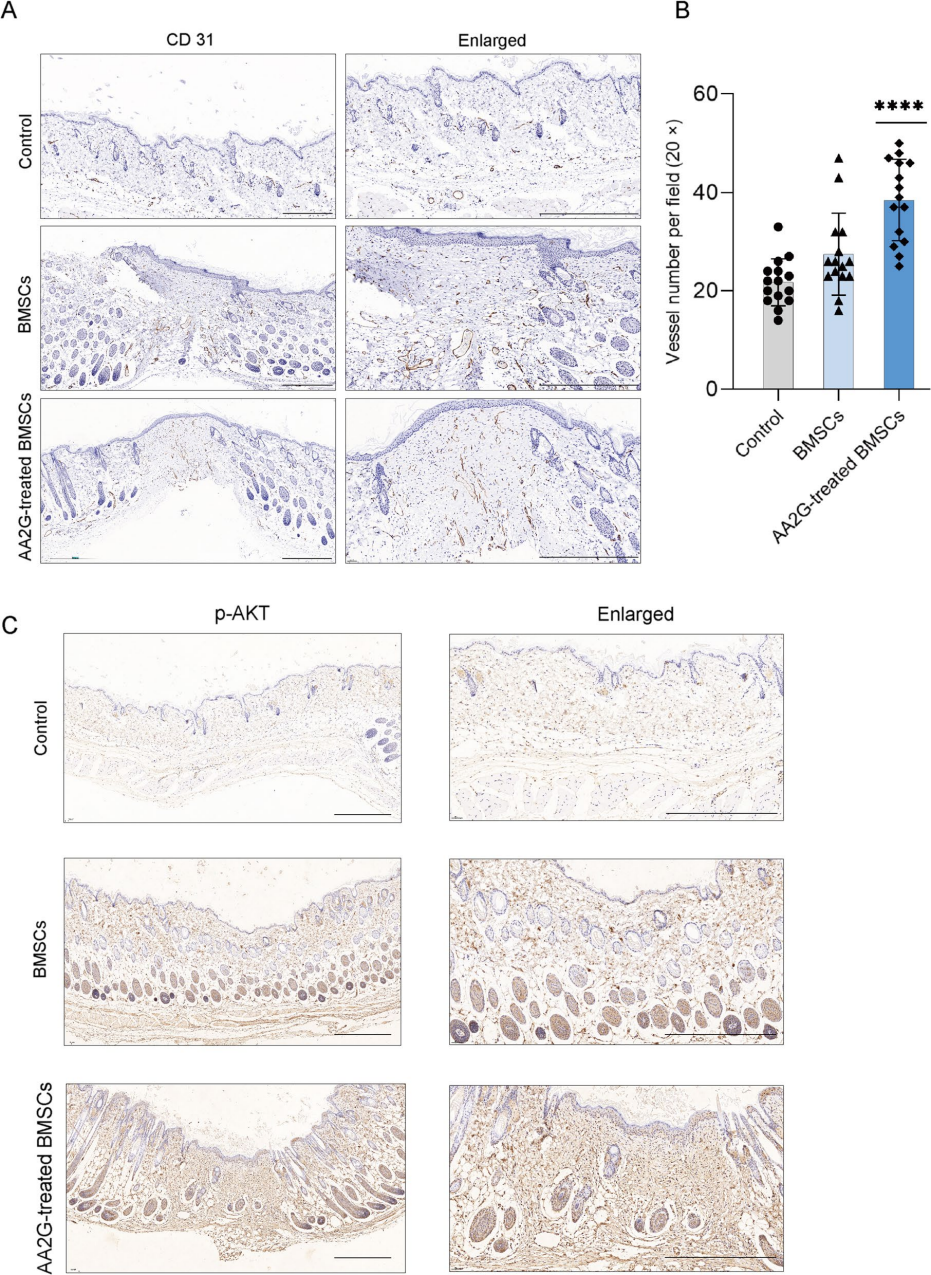
AA2G-treated BMSCs promoted angiogenesis in wound tissues. **a** Representative images of CD31 staining of wounds tissue in control, BMSCs, and AA2G-treated BMSCs groups. Bar = 500 µm. **b** Microvessel density in the wounds was assessed by CD31-positive staining and the number of CD31-positive microvessels per field was calculated (*n* = 15). *P* < 0.0001 based on one-way ANOVA. **c** The p-AKT staining of repaired skin tissue in control, BMSCs, and AA2G-treated BMSCs groups at day 14. Bar = 500 µm

The IHC staining confirmed that the number of new blood vessels was the least in the control group, and the number of new blood vessels increased in the wound tissue after BMSC treatment (Fig. 6a, b). While the number of new blood vessels in the AA2G-treated BMSCs group was the largest among the three groups (Fig. 6a, b). This result further confirmed the effect of AA2G in reinforcing the angiogenesis ability of BMSCs. Similarly, consistent with the results of cell experiments, IHC results showed that the AA2G-treated BMSCs group showed more p-AKT positive cells in the new wound area, further demonstrating the involvement of the AKT pathway in promoting vascularization (Fig. 6c).

### AA2G adjusted methylation of BMSCs

**Fig. 7:**
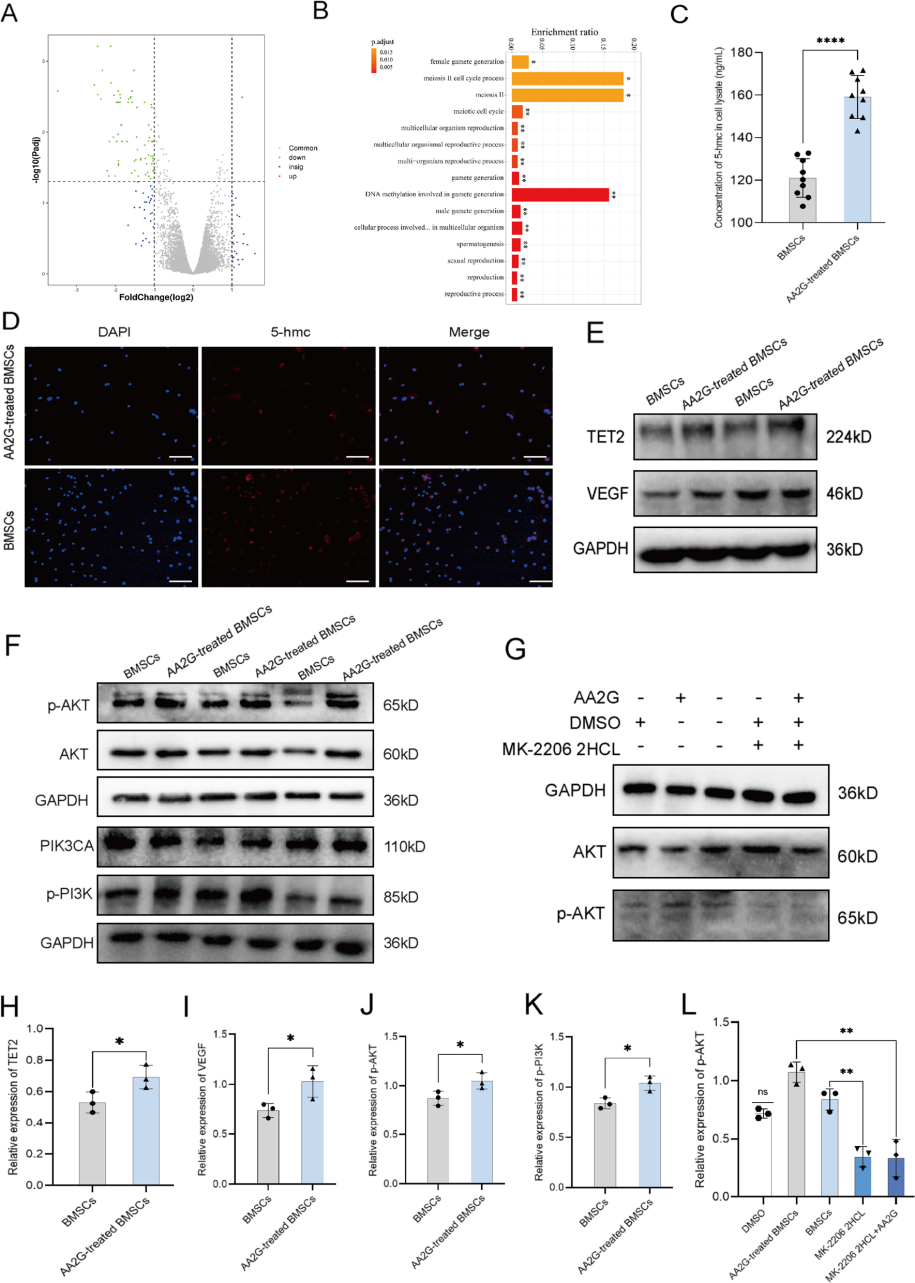
AA2G treatment increased the demethylation of BMSCs. **a** A volcano plot of differentially expressed genes (DEGs). Upregulated DEGs were represented in red and downregulated DEGs were represented in green. **b** After AA2G treatment, the differential genes were mainly concentrated in the biological process, especially in the cell cycle process, meiosis, and DNA methylation was involved in gamete generation. **c** Concentration levels of 5-hmc in BMSCs with or without AA2G treatment (*n* = 9). *P* < 0.0001 based on t-test. **d** Representative images of 5-hmc IF staining showed the demethylation of BMSCs and AA2G-treated BMSCs. **e** Western blot analysis showed the TET2 and VEGF protein expression of BMSCs and AA2G-treated BMSCs. **f** Western blot analysis showed the AKT, p-AKT, PIK3CA, and p-PI3K protein expression of BMSCs and AA2G-treated BMSCs. **g** Western blot analysis showed the AKT and p-AKT protein expression of BMSCs treated with AKT inhibitor. **h** Quantitative analysis of TET2 protein expression of BMSCs (*n* = 3). *P* < 0.05 based on t-test. **i** Quantitative analysis of VEGF protein expression of BMSCs (*n* = 3). *P* < 0.05 based on t-test. **j** Quantitative analysis of p-AKT protein expression of BMSCs (*n* = 3). *P* < 0.05 based on t-test. **k** Quantitative analysis of p-PI3K protein expression of BMSCs (*n* = 3). *P* < 0.05 based on t-test. **l** Quantitative analysis of p-AKT protein expression of AKT inhibitor-treated BMSCs (*n* = 3). *P* < 0.01 based on t-test

To explore the potential biological effect of AA2G-treated BMSCs, we obtained the 77 differentially expressed genes from 6 chip data (GSE106184) in the gene expression omnibus (GEO) database, among which 2 genes were up-regulated and 75 genes were down-regulated (Fig. 7a). Based on GO analysis, these differentially expressed genes were mainly related to biological processes, such as the cell cycle process, meiosis, and DNA methylation involved in gamete generation (Fig. 7b). For experimental verification, ELISA analysis showed the expression level of 5-hmc in the AA2G-treated BMSCs group was increased compared with the BMSCs group, which was consistent with the above dataset analysis (Fig. 7c). In addition, the IF results also showed that the number of 5-hmc-positive cells in the AA2G-treated BMSCs group was higher than that in the BMSCs group, indicating an increased demethylation expression level of BMSCs (Fig. 7d). Because the enzymatic function of the TET2 proteins is to convert 5-mc to 5-hmc, the TET2 levels in BMSCs were measured by western blotting [17]. Compared with the BMSCs group, the protein expression of TET2 in AA2G-treated BMSCs was significantly increased. And, the expression level of VEGF was also significantly increased in the AA2G-treated BMSCs group (Fig. 7e, h, i). Meanwhile, compared with the BMSCs group, the expression levels of p-AKT and p-PI3K in AA2G-treated BMSCs were significantly increased, suggesting that the PI3K/AKT pathway might be activated (Fig. 7f, j, k). Furthermore, MK-2206 2HCL, an AKT pathway inhibitor, significantly reduced the expression level of p-AKT in BMSCs. At the same time, the p-AKT expression level in MK-2206 2HCL combined with AA2G group was significantly lower than that of AA2G alone in BMSCs (Fig. 7g, l). Based on the above results, it can be speculated that AA2G preconditioning might enhance the demethylation process of BMSC by regulating TET2 and up-regulating VEGF expression by activating the PI3K/AKT pathway.

## Discussion

Nowadays, local transplantation of stem cells has emerged as an attractive method to promote wound healing. Here, our study showed that AA2G-treated BMSCs could significantly accelerate the wound healing process in mice, and improve collagen deposition and angiogenesis at wound sites. And in vitro, AA2G treatment promoted the angiogenic ability of BMSCs through activating the PI3K/AKT signaling pathway and enhanced the demethylation process of BMSCs by promoting the TET2 expression. Meanwhile, AA2G-treated BMSCs might positively regulate fibroblast by enhancing the paracrine function. Therefore, the AA2G preconditioning enhanced the therapeutic ability of BMSCs in wound sites both in vitro and in the mouse model.

VitC and its derivatives are known to accelerate wound healing by promoting collagen synthesis and antioxidant action [18]. In specific disease states, relatively low VitC levels in cells or serum result in ineffective wound healing [19]. However, VitC is readily oxidized to dehydroascorbic acid in vitro, which could induce cytotoxicity via consuming the abundant antioxidant glutathione in cells [20]. On the other hand, the cytotoxicity of AA2G is lower than that of VitC, which is due to the lower cellular uptake and oxidation of AA2G [21]. Compared with VitC, AA2G has a series of advantages such as easy acquisition, easy operation and lasting effect, which may promote the function of stem cells [16]. Therefore, we explored the impact of AA2G preconditioning in BMSCs on wound healing. The in vitro results showed that AA2G-preconditioned BMSCs significantly increased proliferation, migration, and angiogenesis, and might promote the function of fibroblasts and vascular endothelial cells through paracrine function. Encouragingly, AA2G-preconditioned BMSCs significantly promoted the wound healing rate accompanied by enhanced collagen disposition and vascularization formation. These results collectively further confirmed the important role of AA2G-preconditioned BMSCs in wound healing.

Wound milieu could cause the initiation and activation of multifarious cooperative signaling pathways, among which the PI3K/AKT signaling pathway has been deeply studied. For instance, Yan et al. revealed that BMSCs stimulated angiogenesis at the wound site by activating the AKT signaling pathway [22]. Besides, velvet antler polypeptide could promote adipose-derived stem cells (ADSCs) angiogenesis by activating the PI3K/Akt/HIF-1α pathway [23]. Xiao et al. demonstrated that ozone oil facilitated wound healing via increasing fibroblast migration and EMT process via PI3K/Akt/mTOR signaling pathway in vivo and in vitro [24]. Our results also showed that the expression level of p-PI3K and p-AKT proteins in the AA2G-treated BMSCs group was significantly higher than that in the BMSCs group. Therefore, it was validated that the PI3K/AKT signal pathways in BMSCs were activated after AA2G preconditioning, which was consistent with the above results.

In addition, epigenetic modification is a key regulatory event in the maintenance of the pluripotency and differentiation of stem cells. TET family can mediate DNA oxidation, which is capable of hydroxylating 5-mc to 5-hmc and then converting 5-hmc to 5-fc and 5-cac. TET enzymes are crucial participants in shaping the methylation landscape in various diseases. The aberrant alteration pattern of 5-hmc can be used as an evaluation indicator for stem cell fate and wound healing disorders. Yang et al. emphasized that the lack of TET1 and TET2 resulted in impaired self-renewal and differentiation, and downregulated the exosome and miRNA release [25]. It confirmed that TET1 and TET2 could maintain BMSC homeostasis by the demethylation of the P2rX7 promoter. Moreover, Li et al. reported that loss of 5-hmc was related to decreased mRNA expression of TET1 and TET2, and loss of 5-hmc was attributed to dysregulation of keratinocyte stem cell dynamics, resulting in the classic psoriatic epidermal structure [26]. In our study, the results of IF and ELISA both demonstrated that AA2G-treated BMSCs possessed higher 5-hmc expression, while western blot results showed the consistent enhanced TET2 and VEGF expression. Hence, it indicated that AA2G was a powerful enhancer of BMSCs wound healing function by influencing TET2 and 5-hmc abundance in BMSCs. Additionally, Yan et al. found that there was a decreased expression of 5-hmc in hADSC derived from older donors, while the pre-treatment of 5-Azacytidine in human adipose-derived stem cells (hADSC) could rejuvenate the hADSC function with enhanced 5-hmc, TET2, and TET3 expression [27]. Therefore, the epigenetic modification of the TET-5-hmc pathway accounts for regulating stem cell fate. The fine-tuned coordination of 5-hmc might be a precise and feasible method in regulating the BMSC-promoted wound healing process.

However, there are still some limitations in this study. First, the recipient site of stem cell transplantation contains exogenous and endogenous stem cells. It is still not quite clear whether the locally injected BMSCs could migrate to distant sites or could recruit the host-derived stem cells, and this needs to be further confirmed in subsequent assays. Then, the components of AA2G-treated BMSC CM are very complex. Therefore, which soluble factors or complex factor signatures play a key role in wound healing deserve further study. Lastly, the wound healing process is not completely the same in mice and humans. The clinical application of AA2G-treated BMSCs in wound healing remains to be further explored.

## Conclusions

In conclusion, our results suggested that AA2G could induce proliferation, angiogenesis and migration of BMSCs in vitro, thereby enhancing the ability of BMSCs on wound healing in mice, which was mediated by enhanced demethylation and activation of PI3K/AKT pathway. Therefore, the AA2G is an important regulator that potentiates the positive function of BMSCs in accelerating wound healing.

## Supplementary Information

**Additional file 1. Figure S1**. FCM analysis gate diagram. (A) Representative images of FCM by BMSCs surface biomarkers staining. (B) Representative images of FCM by cell cycle analysis after BMSCs treated with AA2G for 24 h.

## Data Availability

All datasets generated for this study are included in the manuscript and the supplementary materials.

## Abbreviations

BMSCs: Bone marrow mesenchymal stem cells
AA2G: Ascorbic acid 2-glucoside
AA: Ascorbic acid
VitC: Vitamin C
Chrys: Chrysanthemum morifolium flower extract
mESCs: Mouse embryonic stem cells
hMSCs: Human mesenchymal stem cells
DMEM/F12: Dulbecco’s modified eagle medium/nutrient mixture F-12
FBS: Fetal bovine serum
FCM: Flow cytometry
CM: Conditioned medium
HE: Hematoxylin and eosin
IHC: Immunohistochemistry
IF: Immunofluorescence
BSA: Bovine serum albumin
GO: Gene Ontology
GEO: Gene expression omnibus
ADSCs: Adipose-derived stem cells
hADSCs: Human adipose-derived stem cells
