# Supplementary figures and images for: Ascorbic acid 2-glucoside preconditioning enhances the ability of bone marrow mesenchymal stem cells in promoting wound healing
Source: Stem Cell Res Ther. 2022 Mar 21;13:119. doi: 10.1186/s13287-022-02797-0 (PMC8935805; doi:10.1186/s13287-022-02797-0)

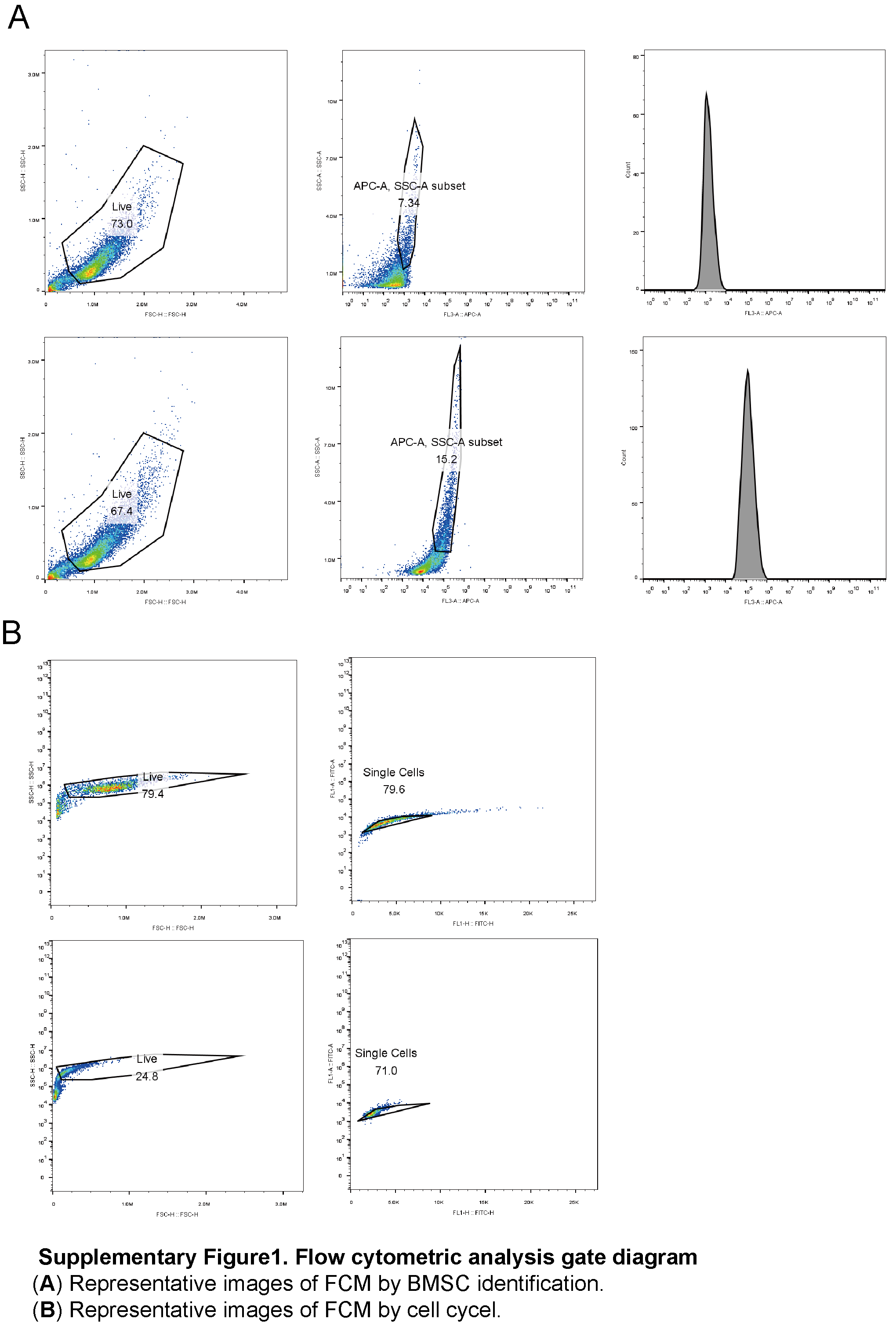

Supplement: Supplementary file 1 — Additional file 1. Figure S1. FCM analysis gate diagram. (A) Representative images of FCM by BMSCs surface biomarkers staining. (B) Representative images of FCM by cell cycle analysis after BMSCs treated with AA2G for 24 h. [file 13287_2022_2797_MOESM1_ESM.tif]
